# Social and Demographic Patterns of Health-Related Internet Use Among Adults in the United States: A Secondary Data Analysis of the Health Information National Trends Survey

**DOI:** 10.3390/ijerph17186856

**Published:** 2020-09-19

**Authors:** Rose Calixte, Argelis Rivera, Olutobi Oridota, William Beauchamp, Marlene Camacho-Rivera

**Affiliations:** 1 Department of Community Health and Social Medicine, CUNY School of Medicine, New York, NY 10031, USA; rcalixte@med.cuny.edu (R.C.); tobi.oridota@gmail.com (O.O.); wbeauch000@citymail.cuny.edu (W.B.); 2 Department of Medicine, Icahn School of Medicine at Mount Sinai, New York, NY 10027, USA; argelis.rivera@mountsinai.org; 3 Department of Community Health Sciences, SUNY Downstate Health Sciences University, Brooklyn, NY 11203, USA

**Keywords:** mobile health, health communication, internet use

## Abstract

National surveys of U.S. adults have observed significant increases in health-related internet use (HRIU), but there are documented disparities. The study aims to identify social and demographic patterns of health-related internet use among U.S. adults. Using data from the Health Information National Trends Survey (HINTS) 4 cycle 3 and HINTS 5 cycle 1, we examined HRIU across healthcare, health information seeking, and participation on social media. Primary predictors were gender, race/ethnicity, age, education, income, and nativity with adjustments for smoking and survey year. We used multivariable logistic regression with survey weights to identify independent predictors of HRIU. Of the 4817 respondents, 43% had used the internet to find a doctor; 80% had looked online for health information. Only 20% had used social media for a health issue; 7% participated in an online health support group. In multivariable models, older and low SES participants were significantly less likely to use the internet to look for a provider, use the internet to look for health information for themselves or someone else, and less likely to use social media for health issues. Use of the internet for health-related purposes is vast but varies significantly by demographics and intended use.

## 1. Introduction

The advancement of technology and ubiquity of the internet has placed individuals at the forefront of their health, making health information more attainable across all demographic groups [1]. Data from Pew Research Center have shown that internet use for racial and ethnic minorities is comparable to non-Hispanic whites [2]. As a result, health dissemination has become more streamlined, and use of the internet has become a popular tool for fostering complex conversations, increasing access to health information, and improving medical outcomes [3,4,5,6,7,8,9,10,11]. For example, systematic reviews of digital health interventions have found clinical benefits among individuals with musculoskeletal conditions and improved quality of life and mental health outcomes for cancer patients [7,8].

However, a recent systematic evidence of web-based interventions targeting self-management across multiple chronic conditions observed differences in the effectiveness of eHealth interventions across various sociodemographic characteristics [5]. Among diabetes studies, women were observed to benefit more from eHealth interventions compared to men; participants with higher levels of education benefited more compared to adults of lower educational backgrounds. Web-based interventions that targeted chronic obstructive pulmonary disease observed greater improvements in clinical outcomes among younger participants compared to older participants. Within this systematic review, several studies examining effect modification of intervention effectiveness by race/ethnicity observed that ethnic minority groups benefited more from diabetes interventions. 

Despite the evidence documenting the effectiveness of internet-based interventions in improving chronic disease outcomes and the wealth of health information available, access and uptake of health information remains a challenge, thus potentially increasing health disparities across race/ethnicity, socioeconomic status, and language [12,13,14,15,16]. In particular, studies have observed gaps in internet use for specific health outcomes (e.g., cancer, cardiovascular disease) across race/ethnicity, age, and gender [17,18,19,20,21,22]. A secondary analysis of data from the Pew Research Center observed that racial and ethnic minority groups were more likely to contribute to COVID-19 content on social media [14]. A previous analysis of health-related internet use among cancer survivors documented lower rates of use among older adults, racial and ethnic minorities, adults with lower levels of education, and adults living in rural areas [13]. Additional studies examining potential explanations for lower rates of eHealth use among particular demographic groups have documented lower rates of trust in health information from internet sources among older adults, racial and ethnic minorities, smokers, and men [20,23,24,25]. Studies focused on internet use among older adults have observed higher levels of frustration in identifying trustworthy sources of health information and visual inaccessibility of information on internet sites [15,23,26,27]. 

While many studies have examined use of the internet for health-related purposes for specific health conditions, gaps remain in examining disparities in use for a broad range of purposes including communication with healthcare professionals, health-related social media use, and general health information seeking [28,29]. Further, few studies have examined disparities in health-related internet use (HRIU) across a variety of demographic and health factors among a nationally representative dataset. The objective of this study was to identify disparities in health-related internet use across social and demographic characteristics and by its intended purpose. Through identifying differences in HRIU, we will contribute evidence to support targeted interventions among specific demographic groups, increasing the potential for acceptability and uptake of internet-based interventions among vulnerable groups. We hypothesized that significant differences in HRIU would emerge based on education, income, age, and gender. In particular, we hypothesized that adults who are younger, female, and of higher socioeconomic status (educational background, income, and home owners) would be more likely to use the internet for health-related purposes across all domains, when compared to participants who were older, male, and of lower socioeconomic background.

## 2. Materials and Methods 

### 2.1. Data Sample

The National Cancer Institute’s Health Information National Trends Survey (HINTS) is a publicly available and nationally representative survey of the non-institutionalized adult population of the United States [30,31]. Data for this study came from the following two HINTS cycles: HINTS 4 cycle 3 (*N* = 3185), collected from September 2013 to December 2013, and HINTS 5 cycle 1 (*N* = 3335), collected from January 2017 to May 2017. The sampling design for the HINTS survey has been well described elsewhere [32,33]. The overall response rate was 35.2% for HINTS 2013 and 32.4% for HINTS 2017. As the analytic sample was derived from de-identified publicly available data, Institutional Review Board approval was not required for this study. 

### 2.2. Measures

To assess differences in eHealth usage, the primary outcomes for this analysis were participants’ responses to 8 HINTS variables that were asked of respondents who answered yes to ever going online to access the internet. The 8 eHealth tasks were divided into 3 domains relevant to health communication (healthcare, health information-seeking, and user-generated content/sharing). These domains have been previously utilized to illustrate trends across eHealth tasks and for the purposes of informing future health communication interventions [32]. Within the healthcare domain, participants were asked to respond “yes” or “no” to the following items: “In the past 12 months, have you used the internet for any of the following reasons: used email or internet to communicate with a doctor; looked for a provider online; bought medicine or vitamins online”. Within the health information seeking domain, participants’ responses to whether they had used the internet to look for health information for themselves or for someone else were included. Within the user-generated content/sharing domain, participants were asked whether or not they had used the internet for the following purposes: visited a social networking site to read and share about medical topics; used email or internet to write in an online diary or blog about any type of health topic; participated in an online support group for people with a similar health or medical issue. 

### 2.3. Analysis

Consistent with prior HINTS studies, primary predictors of interest were gender, race/ethnicity, age, education, income, home ownership, and nativity [28,32]. Consistent with prior HINTS analyses, missing responses were imputed using hot-deck imputation method to preserve the distribution of observed responses [34]. Each observation with missing data was imputed 20 times and the imputed weight was recalibrated to preserve the national representative survey weight. Given previous HINTS studies examining differences in eHealth use among smokers and cancer survivors, all models were adjusted for smoking status, personal cancer history, and family cancer history [13,25,35,36,37,38,39,40]. To measure the change in eHealth usage in different HINTS release, a dummy variable was used to represent the survey year.

We conducted multivariable logistic regression models to identify independent predictors of eHealth usage among a sample of US adults with access to the internet. All analyses were done using SAS (version 9.4, SAS Institute, Inc., Cary, NC, USA) complex survey methods with jackknife replicate weights to compute accurate standard errors, with all analyses weighted to provide nationally representative estimates. We calculated weighted percentages, odds ratios (OR), and 95% confidence intervals (CI) utilizing complete case analyses with listwise deletion for each model. Results with *p*-value ≤ 0.05 were deemed statistically significant.

## 3. Results

### 3.1. Descriptive Results

The results presented in this paper are from the imputed dataset unless otherwise noted with missing category in the result tables. From the two cycles on HINTS data, a total of 4817 respondents were included in the analytic sample. As presented in Table 1, of 48% of respondents identified as female and 47% identified as male. Sixty-five percent of respondents were non-Hispanic White, 9% non-Hispanic Black, 13% Hispanic, and 7% were of another race. Over 74% of the responders have had some college education; nearly 85% were born in the United States. More than half of all participants were between the ages of 35 to 64 years (58.2%). Although 91% of the responders had never been diagnosed with cancer, 67% of responders had family members who had been affected by cancer.

We estimated the odds ratio between each pair of HRIU outcomes to examine how the measures influence one another. Participants who reported that they search for health care providers online were 2.83 times as likely to report that they talk to a provider online (95% CI = 2.4, 3.42, *p* < 0.001) and 1.97 times as likely to report that they buy medicine online (95% CI = 1.60, 2.43, *p* < 0.001). They were also 4.67 times as likely to report that they search for health information for themselves (95% CI = 3.26, 6.69, *p* < 0.001) and 2.69 times as likely to report that they search for health information online for someone else (95% CI = 2.18, 3.34, *p* < 0.001). Additionally, they were 2.16 times as likely to report that they shared contents on social networking sites (95% CI = 1.61, 2.90, *p* < 0.001), 3.34 times as likely to report that they wrote blog (95%CI = 2.23, 5.00, *p* < 0.001), and 3.03 times as likely to report that they participate in online support groups (95% CI = 2.05, 4.48, *p* < 0.001). The strongest association was observed between those who reported that they search for health information for themselves and for someone else (OR = 8.18, 95% CI = 6.07, 11.03, *p* < 0.001). However, there was no association between participants who reported that they search for health information for someone else and those who reported that they wrote blogs.

As displayed in Table 2, in the healthcare domain, 44% of respondents have used the internet to look for a healthcare provider. However, only 35% of responders ever used the internet to communicate with a doctor. Twenty-two percent of participants reported ever purchasing medicine or vitamins online. Within the health information-seeking domain, 80% of respondents have used the internet to seek health information for themselves, while 67% have used the internet to seek health related information for someone else. The user generated content domain was the least commonly reported among participants, with 20% of participants having visited a social networking site to read or share health topics. Seven percent of respondents participated in an online support group for a health-related issue, and 6% of adults reported maintenance of an online diary/blog about any health-related topic.

### 3.2. Multivariable Logistic Regression Results—Healthcare Domain

Adjusted results from the multivariable logistic regression models for the healthcare domain are displayed in Table 3. Responders 35 years of age and older were less likely to use the internet to look for a health care provider compare to responders under 35 (OR = 0.67, 95% CI [0.49, 0.92]). Responders over the age of 65 were less likely to use the internet or email as a method of communication with a doctor/doctor’s office compare to responders under 35 (ages 65–74: OR = 0.60, 95% CI [0.42, 0.85]; >75 y/o: OR= 0.46, 95% CI [0.28, 0.76]). There was no difference in the use of the internet for those between 35 and 64 vs. those under 35. Additionally, there were no age differences in the use of the internet to buy medicine or vitamins online.

No racial differences were observed in using the internet to look for a healthcare provider or to communicate with a provider. However, non-Hispanic Blacks were less likely to buy medicine or vitamins online compare to non-Hispanic Whites (OR = 0.56, 95% CI [0.38, 0.84]). Those with some college education or less were also less likely to look for a healthcare provider online compared to those with a college education or more (<HS OR = 0.59, 95% CI [0.46, 0.75]; some college OR = 0.70, 95% CI [0.56, 0.87]). Those with at most a high school education were less likely to use email or internet to communicate with providers (OR = 0.49, 95% CI [0.37, 0.66]). Responders in the lowest income brackets were also less likely to use email or internet to communicate with providers when compared to respondents with incomes greater than 75,000 USD (<20 k USD: OR = 0.38, 95% CI [0.27, 0.54]; 20–35 k USD OR = 0.32, 95% CI [0.22, 0.45]). There has also been a significant increase in the use of the internet to look for a healthcare provider, communicate with a doctor, and purchase of medicine or vitamins, when comparing HINTS respondents from 2013 to 2017.

### 3.3. Multivariable Logistic Regression Results—Health Information Seeking

As observed in Table 4, respondents aged 50 and older were significantly less likely to use the internet to look for health information for both themselves and others as opposed to those 34 and younger (ages 50–64: OR = 0.47, 95% CI [0.30, 0.75]; 65–74: OR = 0.32, 95% CI [0.19, 0.52]; >75 y/o: OR = 0.21, 95% CI [0.11, 0.38]). However, no statistically significant differences were noted for those between the age 35 and 49 vs. those under 35. Women were significantly more likely to seek health or medical information for someone else as opposed to males (OR = 1.74, 95% CI [1.39, 2.18]). Additionally, participants in the HINTS survey with some college education or less were less likely to look for health information online for themselves (<HS: OR = 0.45, 95% CI [0.33, 0.62]; some college: OR = 0.69, 95% CI [0.49, 0.99]), and those with a high school education or less were less likely to look for health information for someone else compared to those with at least a college degree (OR = 0.67, 95% CI [0.49, 0.93]). Having a household income < 20,000 USD significantly decreases the odds of using the internet to look for information for someone else (OR = 0.55, 95% CI [0.36, 0.86]) vs. income > 75,000 USD. Participants who have never been diagnosed with cancer were less likely to look for health or medical information online for both themselves (OR = 0.55, 95% CI [0.40, 0.76]) and others (OR = 0.70, 95% CI [0.54–0.90]). Not having a family member diagnosed with cancer significantly decreases the odds of using the internet to look for health or medical information for someone else (OR = 0.76, 95% CI [0.59–0.99]). Between 2013 and 2017, there were no significant differences in health seeking information for self or someone else. 

### 3.4. Multivariable Logistic Regression Results—User Generated Content/Sharing Domain

As shown in Table 5, women were significantly more likely to read/share health content on social networking sites (OR = 1.93, 95% CI [1.43, 2.60]) and more likely to participate in an online support group (OR = 1.62, 95% CI [1.05, 2.50]), compared to men. Responders over the age of 50 were less likely to participate in any of the three activities that fall under this domain, when compared to adults in the 18–34 years of age category (visited site for health reason—50–64 y/o: OR = 0.30, 95% CI [0.21, 0.43]; 65–74 y/o: OR = 0.22, 95% CI [0.15, 0.35]; >75 y/o: OR = 0.11, 95% CI [0.05, 0.25]), (diary or blog: OR = 0.37, 95% CI [0.17, 0.79]; OR = 0.25, 95% CI [0.11, 0.57]; OR = 0.20, 95% CI [0.06, 0.59], respectively), (online support: OR = 0.39, 95% CI [0.22, 0.69]; OR = 0.12, 95% CI [0.06, 0.25]; OR = 0.08, 95% CI [0.03, 0.25], respectively). Non-Hispanic Blacks were significantly less likely to visit social networking site to read and share about medical topics compared to non-Hispanic Whites (OR = 0.54, 95% CI [0.37, 0.80]). Respondents with a high school degree or less were less likely to write blogs about any health topic (OR = 0.34, 95% CI [0.18, 0.64]) and participate in online support groups (OR = 0.54, 95% CI [0.33–0.91]) for people with a similar health issue compared to those with a college degree or more. However, participants with some college education were more likely to visit social networking site to read and share about medical topics compared to participants with a college degree or more (OR = 1.51, 95% CI 1.12, 2.03]). Participants who had been diagnosed with cancer were more likely to participate in an online support group (never diagnosed: OR = 0.52, 95% CI [0.31, 0.89]). In addition, respondents who had family members affected by cancer were significantly more likely to read/share health content from social networking sites (OR = 0.67, 95% CI [0.49, 0.91]) than those without a family history of cancer. Former and current smokers were less likely to write in an online diary or blog about any type of health topic than non-smokers. The use of user generated content has not significantly changed over the years.

## 4. Discussion

Our study yielded several important findings with respect to how internet use for health-related purposes differs across a range of sociodemographic and health characteristics. This study also suggests that utilization of the internet for health-related purposes varies broadly by intended use and by modality (e.g., internet sites, social media platforms, blogs and virtual support groups, healthcare platforms). 

Consistent with prior studies analyzing disparities in online health-related use, we observed disparities by age across a range of modalities [40,41,42,43,44]. We observed that use of the internet to look for a healthcare provider and communicate with a healthcare provider decreased among older respondents. These differences may be attributable to differences in health literacy, difficulties and frustration in accessing online information, or trust in health information from online sources [23,24,27,45,46,47,48,49,50]. Consistent with previous studies, we observed that older adults were also less likely to use the internet to locate health-related information for themselves or others [13,20,23,27,46]. Prior studies have observed that older individuals are more likely to access and trust health information from other sources, including friends and families and religious organizations [20,27,49]. Lastly, we observed that older adults were less likely to use social media or online groups for seeking and sharing health information as well as for social support. Although fewer studies have been published exploring disparities in social media engagement using nationally representative samples [29,51,52], additional studies have identified barriers to social media use among older adults including lack of trust, frustration with accessing technology, and preferences towards in-person interactions [26,53]. 

Our study also observed significant differences in HRIU across various indicators of socioeconomic status. Education was consistently associated with HRIU across all three domains of healthcare, health information seeking, and user-generated content and sharing. Consistent with prior studies, participants with lower levels of education were less likely to report use of the internet to communicate with healthcare providers, search for health-related information, and use of social media or online blogs/support groups [24,54]. A potential mechanism explaining the association between education and internet use is the role of health and numeric literacy, which has been well documented in previous studies [54,55,56,57,58,59,60]. Lower levels of household income were also associated with lower likelihood of internet use to communicate with healthcare providers and searching for health information for others. These differences may reflect disparities in overall internet access by broadband or cellular networks [61,62]. 

Turning our attention to racial and ethnic disparities, we observed differences in HRIU between non-Hispanic Blacks and non-Hispanic Whites in the areas of purchasing vitamins or medicines online, as well as in use of the social networking sites to read and share about medical topics. While two prior HINTS studies have explored associations between internet use and vitamin/medication purchases [63,64], neither examined racial and ethnic differences, making this a novel finding. While a recent HINTS study did not observe racial/ethnic disparities in social media use [14,51,65], the study focused on use of specific social media, such as Facebook and Twitter, rather than broader online social networking platforms. We also observed a novel finding examining differences in online healthcare use by nativity status. We found that respondents who were foreign born were significantly more likely to purchase medicines or vitamins online when compared to those who were U.S. born, even after for adjusting for race/ethnicity and socioeconomic status. These findings may reflect differences in medication access that were unaccounted for by our statistical models or preferences for alternative or complementary medicines that may be more readily accessed online [66,67]. 

Our study also identified that personal and familial health factors were associated with several HIRU outcomes across all three domains. Consistent to prior studies, current smokers were less likely to engage in many HRIU activities including using the internet to communicate with healthcare providers and writing in online diaries or blogs. These patterns may reflect lower levels of social engagement and trust in medical sources of health information among smokers compared to never smokers [24,25]. Although we did not observe differences among smokers versus non-smokers with respect to searching for health information online, our findings are consistent with prior studies that have reported widespread use of the internet among smokers [35,39]. Consistent with prior studies, we observed increased HRIU among individuals with a personal or family history of cancer, compared to those without [36,37,38,68,69]. These findings highlight the potential for eHealth and mHealth-based interventions to target the unique needs of cancer survivors [36,70]. 

## 5. Study Strengths and Limitations

Strengths of our study include the exploration of internet use for a variety of health-related purposes (e.g., information seeking, communication) and across various modalities. The use of probability sampling used for HINTS yields results that are nationally representative of non-institutionalized adults in the U.S., extending the geographic limitations from prior studies using community, city, and state-specific samples to survey adult populations. Additional strengths include the use of several HINTS cycles to assess changes in health-related internet use within the U.S. over time, as well as the inclusion of demographic factors, as well as health factors such as smoking status and cancer history. 

Although our research has yielded several important additions to the literature examining the digital divide and health disparities, this study has several limitations that should be considered when interpreting study findings. While we were able to examine disparities between major racial and ethnic groups (e.g., non-Hispanic Blacks, non-Hispanic Whites, and Hispanics), small sample sizes of other groups (Asian and Native American/Pacific Islander) limited our ability to identify potential differences between these groups. Further, we were unable to examine potential variation within racial and ethnic groups by various factors such as nativity or country of origin. While we were able to adjust for health factors such as smoking and cancer history, we were unable to adjust for other health conditions that may influence HRIU (e.g., diabetes, hypertension). In addition, we were unable to ascertain which specific social media sites individuals used (e.g., Facebook, Reddit, Twitter, etc.) within the user-generated content/sharing domain. Lastly, due to the cross-sectional self-reported nature of the HINTS, responses may suffer from recall or misclassification bias or social desirability, and we are unable to make conclusions on causality.

## 6. Conclusions

Our study revealed several important sociodemographic and health factors associated with adult health-related internet use. The strongest predictors were age and education, where increased age was associated with reduced health-related internet use across various outcomes in the healthcare, health information seeking, and user-generated content domains. We also observed that respondents with lower levels of education were less likely to engage in a variety of HRIU activities. These findings are consistent with several previous studies and have been examined in association with barriers of health literacy, trust, and frustration with health information on the internet. While we did not observe many disparities in HRIU by nativity or race/ethnicity, we did observe novel findings in purchasing of medicines/vitamins online. Lastly, our study highlighted the importance of considering health characteristics of individuals, in addition to broad social and demographic factors, when designing internet-based health interventions. Our study observed many patterns of increased HRIU among cancer survivors or those with a family history of cancer, while documenting challenges to reaching smokers through the internet or social media for may HRIU activities. Among cancer survivors, our study provides evidence of acceptability of the internet to improve physician/patient communication, obtain relevant health information, and use of social media to promote social support through participation among online groups. Among current smokers, while lower levels of internet use were observed for identifying health providers and sharing personal health experiences, smokers were just as likely to search for health information on the internet compared to non-smokers, which may provide opportunities to virtually disseminate smoking cessation resources to vulnerable groups. These results indicate that programs and interventions designed to reduce health inequities through use of the internet and m-health need consider a variety of demographic factors and social and health needs to increase acceptability and effectiveness of interventions. These results also highlight that, while internet use and health information seeking may be increasing overall, disparities in access and use among many U.S. adults persist, which may further exacerbate health inequities. 

## Figures and Tables

**Table 1:** Sociodemographic characteristics of Health Information National Trends (HINTS) participants (*N* = 4817).

Sociodemographics	*N*	Unweighted Percent	Weighted Percent (Standard Error)
Gender			
Male	1819	37.76	46.6 (0.67)
Female	2694	55.93	48.37 (0.59)
Missing	304	6.31	5.02 (0.46)
Age Group			
18–34	738	15.32	27.82 (1.07)
35–49	1188	24.66	31.01 (1.12)
50–64	1687	35.02	27.18 (0.60)
65–74	777	16.13	8.09 (0.23)
>75	308	6.39	4.00 (0.21)
Missing	119	2.47	1.91 (0.25)
Race/Ethnicity			
Non-Hispanic White	2890	60.00	64.75 (0.52)
Non-Hispanic Black	579	12.02	8.91 (0.39)
Hispanic	635	13.18	13.21 (0.37)
Other	353	7.33	7.36 (0.28)
Missing	360	7.47	5.78 (0.44)
Education Level			
≤ than High School	892	18.52	24.19 (0.73)
Some College	1477	30.66	35.08 (0.67)
College Graduate or More	2362	49.03	39.50 (0.39)
Missing	86	1.79	1.23 (0.39)
Annual household income (USD)			
Less than 20,000	605	12.56	12.14 (0.79)
20,000 to < 35,000	549	11.4	10.61 (0.84)
35,000 to < 50,000	609	12.64	13.84 (0.95)
50,000 to < 75,000	855	17.75	18.21 (0.80)
>75,000	1766	36.66	37.25 (1.00)
Missing	433	8.99	7.94 (0.71)
US Born			
Yes	4097	85.05	84.78 (0.76)
No	653	13.56	14.19 (0.73)
Missing	67	1.39	1.02 (0.18)
Smoking Status			
Current	598	12.41	15.98 (0.98)
Former	1282	26.61	23.97 (0.87)
Never	2900	60.2	59.56 (1.17)
Missing	37	0.77	0.49 (0.13)
Home Ownership			
Own	3303	68.57	62.14 (1.01)
Rent or occupied without rent	1357	28.17	35.33 (1.02)
Missing	157	3.26	2.53 (0.30)
Ever diagnosed as having cancer?			
Yes	674	13.99	7.80 (0.20)
No	4109	85.3	91.70 (0.23)
Missing	34	0.71	0.51 (0.13)
Any family members ever had cancer?			
Yes	3302	68.55	67.04 (0.97)
No	1373	28.5	30.44 (0.93)
Missing	142	2.95	2.52 (0.31)

**Table 2:** Frequency and percent of eHealth tasks among HINTS participants.

eHealth Task	*N*	Unweighted Percent	Weighted Percent (Standard Error)
Ever looked for information about health or medical topics from any source?			
Yes	4197	87.13	84.35 (0.99)
No	576	11.96	14.59 (0.96)
Healthcare Domain			
In the past 12 months, used email or Internet to communicate with a doctor or doctor’s office?			
Yes	1769	36.72	35.48 (0.94)
No	3013	62.55	63.95 (0.96)
In the past 12 months, bought medicine or vitamins online?			
Yes	1117	23.19	22.17 (1.00)
No	3641	75.59	77.02 (1.02)
In the past 12 months, used the Internet to look for a health care provider?			
Yes	1983	41.17	43.55 (1.17)
No	2769	57.48	55.47 (1.20)
Health Information Seeking Domain			
In the past 12 months, used the Internet to look for health or medical information for self?			
Yes	3864	80.22	79.96 (1.03)
No	922	19.14	19.46 (1.04)
In the past 12 months, used the Internet to look for health or medical information for someone else?			
Yes	3142	65.23	67.46 (1.09)
No	1639	34.03	32.09 (1.12)
User Generated Content/Sharing Domain			
In the past 12 months, participated in an online support group for people with a similar health or medical issue?			
Yes	335	6.95	7.30 (0.52)
No	4454	92.46	92.08 (0.55)
In the past 12 months, visited a social networking site to read and share about medical topics?			
Yes	856	17.77	20.21 (0.98)
No	3933	81.65	79.08 (1.01)
In the past 12 months, wrote in an online diary or blog about any type of health topic?			
Yes	255	5.29	6.06 (0.57)
No	4524	93.92	93.20 (0.60)

**Table 3:** Results of multivariable logistic regression models for healthcare domain outcomes (*N* = 4817).

Sociodemographics	Look for Health Care Provider		Used Email or Internet to Communicate with Doctor		Bought Medicine or Vitamins Online	
	OR	95% CI	OR	95% CI	OR	95% CI
Gender (ref: Male)						
Female	1.16	0.96, 1.41	1.14	0.91, 1.43	1.25	0.99, 1.58
Age Group (ref: 18–34)						
35–49	0.67	0.49, 0.92 *	1.08	0.79, 1.47	0.84	0.61, 1.17
50–64	0.49	0.36, 0.65 ***	0.82	0.61, 1.10	0.80	0.55, 1.17
65–74	0.27	0.18, 0.40 ***	0.60	0.42, 0.85 **	0.73	0.47, 1.11
>75	0.16	0.09, 0.28 ***	0.46	0.28, 0.76 **	0.69	0.42, 1.16
Race/Ethnicity (ref: non-Hispanic White)						
Non-Hispanic Black	1.27	0.91, 1.78	0.91	0.61, 1.37	0.56	0.38, 0.84 **
Hispanic	1.03	0.78, 1.34	1.01	0.76, 1.34	0.87	0.65, 1.16
Other	1.41	0.94, 2.11	0.85	0.60, 1.21	0.97	0.65, 1.45
Education Level (ref: College Graduate or more)						
≤than High School	0.59	0.46, 0.75 ***	0.49	0.37, 0.66 ***	0.87	0.62, 1.21
Some College	0.70	0.56, 0.87 **	0.83	0.67, 1.04	1.02	0.77, 1.36
Annual household income (USD) (ref: >75,000)						
Less than 20,000	0.68	0.45, 1.03	0.38	0.27, 0.54 ***	0.35	0.21, 0.59 ***
20,000 to < 35,000	0.77	0.53, 1.13	0.32	0.22, 0.45 ***	0.38	0.24, 0.60 ***
35,000 to < 50,000	0.71	0.50, 1.01	0.54	0.38, 0.80 ***	0.73	0.49, 1.10
50,000 to < 75,000	0.88	0.66, 1.16	0.65	0.50, 0.84 **	0.64	0.49, 0.85 **
US Born (ref: Born in US)						
No	1.24	0.91, 1.70	1.32	0.94, 1.83	1.81	1.25, 2.61 **
Smoking Status (ref: Never Smoker)						
Current	0.70	0.50, 0.99 *	0.91	0.65, 1.29	0.71	0.46, 1.09
Former	0.81	0.65, 1.01	1.00	0.81, 1.23	1.04	0.82, 1.31
Home Ownership (ref: Own)						
Rent or occupied without rent	1.15	0.87, 1.51	1.31	1.03, 1.66 *	0.79	0.60, 1.05
Ever diagnosed as having cancer (ref: Yes, history of cancer)						
No	0.84	0.66, 1.09	0.66	0.51, 0.86 **	0.86	0.61, 1.20
Any family members ever had cancer (ref: Yes, history of cancer)						
No	0.73	0.59, 0.90 **	0.94	0.74, 1.20	1.18	0.88, 1.58
Survey Release (ref: HINTS 4 cycle 3)						
HINTS 5 cycle 1	1.53	1.19, 1.98 ***	1.65	1.26, 2.15 ***	1.40	1.05, 1.89 *

The results were obtained using a multivariable logistic regression model. We present the result using OR (95%CI). * p-value ≤ 0.05, ** p-value ≤ 0.01, and *** p-value ≤ 0.001.

**Table 4:** Results of multivariable logistic regression models for health information seeking domain outcomes (*N* = 4817).

Sociodemographics	Look for Health or Medical Information for Self		Look for Health or Medical Information for Someone Else	
	OR	95% CI	OR	95% CI
Sex (ref: Male)				
Female	1.14	0.87, 1.49	1.74	1.39, 2.18 ***
Age Group (ref: 18–34)				
35–49	0.77	0.48, 1.25	1.06	0.75, 1.50
50–64	0.47	0.30, 0.75 **	0.62	0.45, 0.85 **
65–74	0.32	0.19, 0.52 ***	0.33	0.22, 0.48 ***
>75	0.21	0.11, 0.38 ***	0.29	0.16, 0.51 ***
Race/Ethnicity (ref: non-Hispanic White)				
Non-Hispanic Black	0.87	0.56, 1.34	0.85	0.60, 1.19
Hispanic	0.87	0.58, 1.29	0.87	0.66, 1.16
Other	0.95	0.58, 1.55	1.31	0.91, 1.87
Education Level (ref: College Graduate or more)				
≤than High School	0.45	0.33, 0.62 ***	0.67	0.49, 0.93 *
Some College	0.69	0.49, 0.99 *	0.95	0.72, 1.25
Annual household income (USD) (ref: >75,000)				
Less than 20,000	0.67	0.37, 1.23	0.55	0.36, 0.86 **
20,000 to < 35,000	0.67	0.43, 1.06	0.73	0.47, 1.15
35,000 to < 50,000	1.05	0.67, 1.63	1.05	0.73, 1.49
50,000 to < 75,000	1.03	0.72, 1.47	0.79	0.60, 1.05
US Born (ref: Born in US)				
No	0.89	0.60, 1.32	1.09	0.77, 1.55
Smoking Status (ref: Never Smoker)				
Current	0.71	0.46, 1.09	1.05	0.72, 1.53
Former	0.95	0.71, 1.27	1.01	0.79, 1.29
Home Ownership (ref: Own)				
Rent or occupied without rent	1.18	0.85, 1.64	0.97	0.74, 1.26
Ever diagnosed as having cancer (ref: Yes, history of cancer)				
No	0.55	0.40, 0.76 ***	0.70	0.54, 0.90 **
Any family members ever had cancer (ref: Yes, history of cancer)				
No	0.81	0.61, 1.07	0.76	0.59, 0.99 *
Survey Release (ref: HINTS 4 cycle 3)				
HINTS 5 cycle 1	1.23	0.86, 1.75	1.03	0.78, 1.35

The results were obtained using a multivariable logistic regression model. We present the result using OR (95%CI). * *p*-value ≤ 0.05, ** *p*-value ≤ 0.01, and *** *p*-value ≤ 0.001.

**Table 5:** Results of multivariable logistic regression models for the user-generated content/sharing domain outcomes (*N* = 4817).

Sociodemographics	Visited a Social Networking Site to Read and Share about Medical Topics		Wrote in an Online Diary or Blog about Any Type of Health Topic		Participated in an Online Support Group for People with a Similar Health or Medical Issue	
	OR	95% CI	OR	95% CI	OR	95% CI
Gender (ref: Male)						
Female	1.93	1.43, 2.60 ***	0.94	0.59, 1.49	1.62	1.05, 2.50 *
Age Group (ref: 18–34)						
35–49	0.82	0.56, 1.19	0.69	0.41, 1.17	0.79	0.52, 1.20
50–64	0.30	0.21, 0.43 ***	0.37	0.17, 0.79 **	0.39	0.22, 0.69 **
65–74	0.22	0.15, 0.35 ***	0.25	0.11, 0.57 **	0.12	0.06, 0.25 ***
>75	0.11	0.05, 0.25 ***	0.20	0.06, 0.59 **	0.08	0.03, 0.25 ***
Race/Ethnicity (ref: non-Hispanic White)						
Non-Hispanic Black	0.54	0.37, 0.80 **	0.64	0.29, 1.42	0.66	0.34, 1.29
Hispanic	0.77	0.56, 1.05	0.66	0.33, 1.30	0.86	0.45, 1.64
Other	0.82	0.49, 1.38	0.73	0.34, 1.55	1.17	0.42, 3.28
Education Level (ref: College Graduate or more)						
≤than High School	1.35	0.95, 1.92	0.34	0.18, 0.64 ***	0.54	0.33, 0.91 *
Some College	1.51	1.12, 2.03 **	0.87	0.49, 1.55	0.80	0.51, 1.24
Annual household income (USD) (ref: >75,000)						
Less than 20,000	1.09	0.71, 1.67	1.37	0.70, 2.67	1.71	0.85, 3.45
20,000 to < 35,000	0.96	0.65, 1.43	1.89	0.95, 3.73	1.45	0.85, 2.47
35,000 to < 50,000	1.03	0.74, 1.42	0.74	0.37, 1.51	1.41	0.79, 2.51
50,000 to < 75,000	0.97	0.69, 1.36	1.91	1.11, 3.31 *	1.03	0.66, 1.60
US Born (ref: Born in US)						
No	1.31	0.88, 1.95	1.16	0.61, 2.22	0.85	0.49, 1.48
Smoking Status (ref: Never Smoker)						
Current	0.98	0.66, 1.48	0.49	0.25, 0.96 *	1.44	0.73, 2.82
Former	0.87	0.64, 1.20	0.56	0.34, 0.92 *	1.27	0.85, 1.90
Home Ownership (ref: Own)						
Rent or occupied without rent	1.03	0.77, 1.37	1.28	0.80, 2.05	0.78	0.54, 1.13
Ever diagnosed as having cancer (ref: Yes, history of cancer)						
No	0.72	0.51, 1.01	0.64	0.35, 1.17	0.52	0.31, 0.89 *
Any family members ever had cancer (ref: Yes, history of cancer)						
No	0.67	0.49, 0.91 *	1.06	0.67, 1.70	0.95	0.63, 1.45
Survey Release (ref: HINTS 4 cycle 3)						
HINTS 5 cycle 1	0.85	0.62, 1.17	1.24	0.68, 2.24	1.31	0.74, 2.33

The results were obtained using a multivariable logistic regression model. We present the result using OR (95%CI). * *p*-value ≤ 0.05, ** *p*-value ≤ 0.01, and *** *p*-value ≤ 0.001.
